# Demographics and mortality trends of valvular heart disease in older adults in the United States: Insights from CDC-wonder database 1999–2019

**DOI:** 10.1016/j.ijcrp.2024.200321

**Published:** 2024-08-17

**Authors:** Eman Ali, Yusra Mashkoor, Fakhar Latif, Fnu Zafrullah, Waleed Alruwaili, Sameh Nassar, Karthik Gonuguntla, Harshith Thyagaturu, Mohammad Kawsara, Ramesh Daggubati, Yasar Sattar, Muhammad Sohaib Asghar

**Affiliations:** a Dow University of Health Sciences, Karachi, Pakistan; b Department of Cardiology, Ascension Borgess Hospital/Michigan State University, MI, USA; c Department of Internal Medicine, West Virginia University, Morgantown, WV, USA; d Department of Cardiology, West Virginia University, Morgantown, WV, USA; e Division of Nephrology and Hypertension, Mayo Clinic, Rochester, MN, USA

**Keywords:** Valvular heart disease, Epidemiology, Mortality, Trend, AAMR

## Abstract

**Background:**

Valvular heart disease (VHD) represents a spectrum of cardiac conditions, including valvular stenosis, valvular regurgitation, or mixed lesions affecting single or multiple valves. The severity of VHD has emerged as a major cause of cardiovascular (CV) morbidity and mortality among the older population in the United States (U.S).

**Objective:**

To evaluate temporal trends in mortality associated with VHD in the elderly U.S population between 1999 and 2019.

**Methods:**

We utilized the CDC WONDER database for VHD mortality in adults ≥75 from 1999 to 2019, using ICD-10 codes. Age-adjusted mortality rates (AAMR) per 100,000 people with associated annual percentage change (APC) were calculated. Joinpoint regression was used to assess the overall trends and trends for demographic, geographic, and type of valvular disease subgroups.

**Results:**

A total of 666,765 VHD deaths in older adults from 1999 to 2019 was identified, with an initial decline in AAMR until 2007 with an APC: 0.62, 95 % CI (−1.66-0.33), stability until 2014, and a significant decrease until 2019 (APC: 1.47, 95 % CI [-2.24-1.04], P < 0.0001). Men consistently had higher AAMRs compared to women (overall AAMR men: 173.6; women: 138.2). The AAMRs were found to be highest in the White (166.5), followed by American Indian or Alaska Native population at (93.8) Hispanic or Latino at (80.7), Black or African American populations at (74.1) and lastly Asian or Pacific Islander (73.4). Non-metropolitan areas manifested higher AAMRs for deaths related to VHD than metropolitan areas (overall AAMRs 160.5 vs 149.5) respectively. State-wide AAMRs varied, with the highest in Vermont at 324.2 (95 % CI [313.0–335.4], P < 0.0001) and the lowest in Mississippi at 88.0 (95 % CI [85.0–91.0], P < 0.0001). Non-rheumatic and aortic valve disorders in adults ≥75 years had higher mortality rates compared to rheumatic or mitral valve conditions in those <75 years.

**Conclusion:**

Our study showed a decline in U.S. VHD mortality from 1999 to 2019 but found persistent disparities by gender, race, age, region, and VHD type. Targeted policies for prevention and early diagnosis are needed to address these inequalities.

## 1 Introduction

Valvular heart disease (VHD) is a group of cardiac conditions that involve abnormalities, such as stenosis, regurgitation, or mixed lesions of both, in one or more heart valves, including the mitral, tricuspid, aortic, and pulmonary valves [1]. These diseases place a hemodynamic burden on the ventricles, eventually manifesting as ventricular muscle dysfunction and congestive heart failure (CHF) [2]. As such, valvular heart diseases are a progressively growing cause of cardiovascular (CV) morbidity and mortality in the United States (U.S).

Life expectancy in the U.S is increasing, and it is estimated that the population aged over 80 years will triple, growing from 11.4 million in 2010 to 32.4 million by 2050 [3]. The prevalence of VHD increases with age, resulting in a growing concern about an increase in projected valvar heart disease burden in the coming decades. Around 2.5 % of the American population suffered from valvular heart disease in 2014, which was more common in the elderly [1]. Furthermore, about 13 % of people born before 1943 have VHD [1]. With such staggering statistics, the efforts to mitigate the public health impact of VHD must be accelerated.

Effective management of VHD in elderly individuals requires a collaborative multidisciplinary team involving anaesthesiologists, interventional and imaging cardiologists, and cardiac surgeons. Historically, surgery has been the cornerstone of the treatment; however, postoperative clinical outcomes are heavily influenced by concomitant comorbidities, such as chronic kidney disease (CKD) and diabetes mellitus (DM) [4]. Minimally invasive approaches have been introduced to alleviate the shortcomings of surgery, like transcatheter valve repairs, which have better prognoses and fewer complications [5]. Recently, there have been notable shifts in healthcare practices, diagnostic capabilities, and therapeutic interventions. Understanding how these changes have influenced the demographics and outcomes of VHD-related mortality is of paramount importance for healthcare providers, researchers, and policymakers to provide optimal care for this vulnerable population. Consequently, this study was conducted with the objective of retrospectively evaluating the temporal trends in mortality associated with VHD in the elderly population of the U.S between 1999 and 2019.

## 2 Methods

### 2.1 Data sources

Our analysis involved the data from Centers for Disease Control and Prevention, Wide-Ranging Online Data for Epidemiologic Research (CDC WONDER) database. Multiple Cause-of-Death Public Use Record death certificates were used to analyze deaths in individuals ≥75 years of age in which valvular heart disease was mentioned as either contributing or underlying cause of death [6,7]. We used the following International Classification of the Diseases, Tenth Revision (ICD-10) codes to identify valvular heart disease-related cases: I08.0 (Disorders of both mitral and aortic valves); I08.1 (Disorders of both mitral and tricuspid valves); I08.2 (Disorders of both aortic and tricuspid valves); I08.3 (“Combined disorders of mitral, aortic and tricuspid valves”); I08.8 (“Other multiple valve diseases”); I08.9 (“Multiple valve disease, unspecified”); I34.0 (“Mitral (valve) insufficiency”); I34.1 (“Mitral (valve) prolapse”); I34.2 (“Nonrheumatic mitral (valve) stenosis”); I34.8 (“Other nonrheumatic mitral valve disorders”); I34.9 (“Nonrheumatic mitral valve disorder, unspecified”); I35.0 (“Aortic (valve) stenosis”); I35.1 (“Aortic (valve) insufficiency”); I35.2 (“Aortic (valve) stenosis with insufficiency”); I35.8 (“Other aortic valve disorders”); I35.9 (“Aortic valve disorder, unspecified”); I36.0 (“Nonrheumatic tricuspid (valve) stenosis”); I36.1 (“Nonrheumatic tricuspid (valve) insufficiency”); I36.2 (“Nonrheumatic tricuspid (valve) stenosis with insufficiency”); I36.8 (“Other nonrheumatic tricuspid valve disorders”); I36.9 (“Nonrheumatic tricuspid valve disorder, unspecified”); I37.0 (“Pulmonary valve stenosis”); I37.1 (“Pulmonary valve insufficiency”); I37.2 (“Pulmonary valve stenosis with insufficiency”); I37.8 (“Other pulmonary valve disorders”); I37.9 (“Pulmonary valve disorder, Unspecified”) [6,7].

### 2.2 Ethics committee

Institutional review board approval was not sought since the data was publicly available, anonymized government data. Additionally, the study was conducted in accordance with the STROBE (Strengthening the Reporting of Observational Studies in Epidemiology) guidelines.

### 2.3 Data extraction

The number of valvular heart disease-related deaths and population size were extracted from 1999 to 2019. The data on age, sex, race and ethnicity, region, and state were also abstracted. We then selected patients aged ≥75 years and divided them into 10-year age groups. Thereafter, we categorized older patients based on sex and studied individual mortality trends for both sexes. For race, patients were stratified into White, Black, Hispanic, Asian/Pacific Islander, and American Indian/Alaskan Native. In addition, a sub-group analysis for rheumatic versus non-rheumatic valvular disorders and aortic versus mitral valvular diseases was also performed. Moreover, mortality trends were also compared with younger patients with valvular disorders by stratifying patients into 2 groups: patients aged ≥75 years and patients aged <75 years. The population was further stratified into metropolitan and non-metropolitan areas using the National Center for Health Statistics Urban-Rural Classification Scheme [8].

### 2.4 Statistical analysis

The number of valvular heart disease-related deaths was divided by the total corresponding population to calculate the crude death rates for individual years. The annual mortality rates were calculated per 100,000 population, and their corresponding 95 % confidence intervals (CIs) were also determined [9]. The mortality rates were adjusted for age by standardizing to the year 2000 US standard population. Temporal trends in mortality were examined to deduce changes in slope using Joinpoint Regression Program version 4.7.0.0, which models consecutive linear segments on a log scale connected by Joinpoints, where the segments converge [10]. Annual percentage changes (APC) and Average Annual Percentage Changes (AAPCs) were calculated, with their corresponding 95 % CIs for the line segments linking a Joinpoint. Slopes were considered increasing or decreasing if the estimated slope differed significantly from zero. The statistical significance was determined by 2-sided t-testing (P = 0.05).

## 3 Results

### 3.1 Annual trends for VHD-related AAMR

Between 1999 and 2019, a total of 666,765 VHD-related deaths occurred in the study population (Table 1). Over the years, a decline in AAMRs for VHD-related deaths among older adults has been observed, with AAMR 158.8 (95 % CI: 156.9–160.8) in 1999 and 142.6 (95 % CI: 141.0–144.1) in 2019. Notably, overall AAMR for VHD-related deaths exhibited a significant reduction from 1999 to 2007 with an APC of −0.62 [95 % CI: 1.66 to −0.33]. Following this period, AAMRs remained stable between 2007 and 2014 (APC: 0.21 [95 % CI: 0.11 to 1.23]). Subsequently, from 2014 to 2019, another significant decline in AAMRs was observed (APC: 1.47 [95 % CI: 2.24 to −1.04]). Demographic characteristics, including the number of deaths and AAMRs, with APCs and AAPCs among the elderly with VHD-related mortality, have been highlighted in Table 1, Table 2, and Supplementary Table 1.

**Table 1:** Demographic characteristics of deaths due to valvular heart disease among the elderly in the USA from 1999 to 2019.

Variable	Death (n)	AAMR
Overall	666,765	151.5 (151.1, 151.8)
**Sex**		
Males	276,068	173.6 (172.9–174.2)
Females	390,697	138.2 (137.8–138.6)
**Race/Ethnicity**		
American Indian or Alaska Native	1576	93.8 (89.1, 98.4)
Asian or Pacific Islander	10,290	73.4 (71.9, 74.8)
Black or African American	24,476	74.1 (73.2, 75.1)
White	608,575	166.5 (166.0, 166.9)
Hispanics	20,851	80.7 (79.6, 81.8)
**Metropolitan/non-metropolitan**		
Metropolitan	540,470	149.5 (149.1, 149.9)
Non-metropolitan	126,295	160.5 (159.6, 161.3)
**Rheumatic/Non-Rheumatic Valve Disease**		
Rheumatic Valve Disease	55,568	12.9 (12.8, 13.0)
Non-Rheumatic Valve Disease	105,521	24.3 (24.1, 24.4)
**Aortic/Mitral Valve Disorder**		
Aortic Valve Disorder	558,629	126.7 (126.3, 127)
Mitral Valve Disorder	129,459	30.0 (29.8, 30.1)
**Younger than 75 Years/Older than 75 Years**		
Younger than 75 Years	172,372	23.8 (23.7, 23.9)
Older than 75 Years	666,765	1515.0 (1511.4, 1518.7)

**Table 2:** Annual percentage changes (APCs) and average annual percentage changes (AAPCs) in valvular heart diseases- related mortality rate among the elderly in the USA from 1999 to 2019.

Variable	Trend Segment	Year Interval	APC (95 % CI)	AAPC (95 % CI)
Overall	1	**1999–2007**	−0.62* (−1.66, −0.33)	−0.55* (−0.67, −0.45)
	2	**2007–2014**	0.21 (−0.11, 1.23)	
	3	**2014–2019**	−1.47 (−2.24, −1.04)	
Men	1	**1999–2006**	−0.68* (−1.44, −0.29)	−0.51* (−0.62, −0.41)
	2	**2006–2014**	0.47* (0.17, 1.19)	
	3	**2014–2019**	−1.83* (−2.43, −1.34)	
Women	1	**1999–2015**	−0.43 (−0.54, 0.39)	−0.62* (−0.77, −0.43)
	2	**2015–2019**	−1.36* (−3.09, −0.61)	
White	1	**1999–2007**	−0.42* (−1.43, −0.12)	−0.25* (−0.38, −0.16)
	2	**2007–2014**	0.57* (0.22, 1.66)	
	3	**2014–2019**	−1.13* (−1.94, −0.65)	
Black or African American	1	**1999–2009**	−1.09 (−4.38, 1.33)	−0.49* (−0.83, −0.10)
	2	**2009–2019**	0.11 (−0.81, 3.41)	
American Indian or Alaska Native	1	**1999–2011**	1.29 (−0.13, 13.89)	−0.50 (−1.64, 1.12)
	2	**2011–2019**	−3.13* (−13.17,-0.82)	
Hispanic or Latino	1	**1999–2019**	−0.83* (−1.12, −0.49)	−0.83* (−1.12, −0.49)
Asian or Pacific Islander	1	**1999–2019**	−1.25* (−1.67, −0.72)	−1.25* (−1.67, −0.72)
Nonmetropolitan areas	1	**1999–2015**	0.33* (0.15, 1.17)	0.01 (−0.23, 0.28)
	2	**2015–2019**	−1.26* (−4.15, −0.02)	
Metropolitan area	1	**1999–2007**	−0.74* (−1.83, −0.44)	−0.65* (−0.77, −0.56)
	2	**2007–2014**	0.10 (−0.22, 1.12)	
	3	**2014–2019**	−1.56* (−2.32, −1.11)	
Rheumatic Valve Disease	1	**1999–2004**	−5.38* (−7.63, −3.96)	−1.11* (−1.34, −0.91)
	2	**2004–2012**	−1.05 (−5.85, 0.44)	
	3	**2012–2017**	0.83 (−1.56, 1.86)	
	4	**2017–2019**	5.01* (2.01, 6.92)	
Non-Rheumatic Valve Disease	1	**1999–2012**	−4.77* (−5.16, −4.56)	−2.58* (−2.72, −2.44)
	2	**2012–2017**	2.42 (−4.75, 3.28)	
	3	**2017–2019**	−0.32 (−2.02, 1.87)	
Mitral Valve Disorders	1	**1999–2004**	−5.50* (−7.40, −4.72)	−2.65* (−2.79, −2.50)
	2	**2004–2012**	−3.63* (−4.18, −2.12)	
	3	**2012–2019**	0.61* (0.06, 1.38)	
Aortic Valve Disorders	1	**1999–2006**	−0.46* (−1.56, −0.07)	−0.38* (−0.49, −0.28)
	2	**2006–2013**	0.84* (0.47, 1.89)	
	3	**2013–2019**	−1.71* (−2.18, −1.31)	
Younger than 75 Years	1	**1999–2008**	−3.09* (−3.88, −2.78)	−1.10* (−1.19, −0.99)
	2	**2008–2012**	1.4166 (−2.74, 1.38)	
	3	**2012–2019**	1.72* (1.35, 2.36)	
Older than 75 Years	1	**1999–2007**	−0.6168* (−1.67, −0.33)	−0.54* (−0.66, −0.46)
	2	**2007–2014**	0.21 (−0.10, 1.20)	
	3	**2014–2019**	−1.48* (−2.23, −1.03)	

### 3.2 VHD-related AAMR stratified by sex

Throughout the study, it was observed that older men consistently had higher AAMRs compared to older women (overall AAMR men: 173.6 [95 % CI: 172.9–174.2]; overall AAMR women: 138.2 [95 % CI: 137.8–138.6]). Specifically, in 1999, the AAMR for men was 180.1 (95 % CI: 176.5–183.7), which gradually decreased to 168.8 (95 % CI: 165.6–172.0) in 2006 (APC: 0.68 [95 % CI: 1.44 to −0.29]). However, this was followed by a slight increase until 2014 (APC: 0.47 [95 % CI: 0.17 to 1.19]). Subsequently, there was a notable steep decline in AAMRs from 2014 to 2019 (APC: 1.83 [95 % CI: 2.43 to −1.34]), and at the end of the study period, the AAMR of 163.4 (95 % CI: 160.8–166.0) was noted in men.

**Fig. 1:**
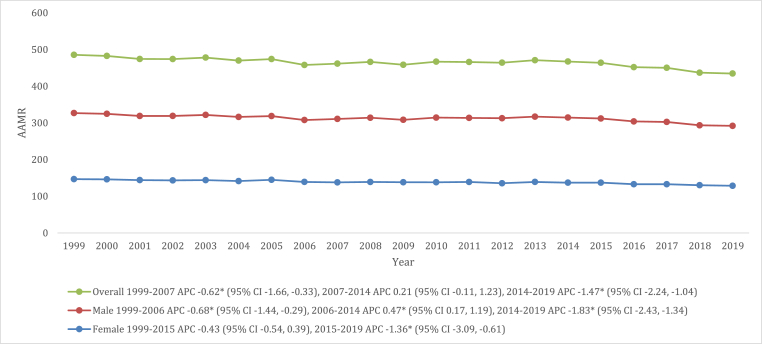
VHD-related mortality Trends in older adult population and stratified for sex in 1999–2019.

On the other hand, the AAMR for women in 1999 was 146.8 (95 % CI: 144.5–149.1), and after which there was a period of stability until 2015 (APC: 0.43 [95 % CI: 0.54 to 0.39). Subsequently, a statistically significant decrease was observed from 2015 to 2019 (APC: 1.36 [95 % CI: 3.09 to −0.61]), and at the end of the study period, the AAMR of women was 128.7 (95 % CI: 126.8–130.5) (Fig. 1 and Supplementary Table 2).

### 3.3 VHD-related AAMR stratified by race/ethnicity

When stratified by race/ethnicity, variations in AAMRs for VHD-related deaths were evident. The AAMRs were found to be highest in the White population with a rate of 166.5 (95 % CI: 166.0–166.9), followed by American Indian or Alaska Native population at 93.8 (95 % CI: 89.1–98.4), Hispanic or Latino at 80.7 (95 % CI: 79.6–81.8), and Black or African American populations at 74.1 (95 % CI: 73.2–75.1). The lowest AAMRs were observed in the Asian or Pacific Islander populations, with a rate of 73.4 (95 % CI: 71.9–74.8).

**Fig. 2:**
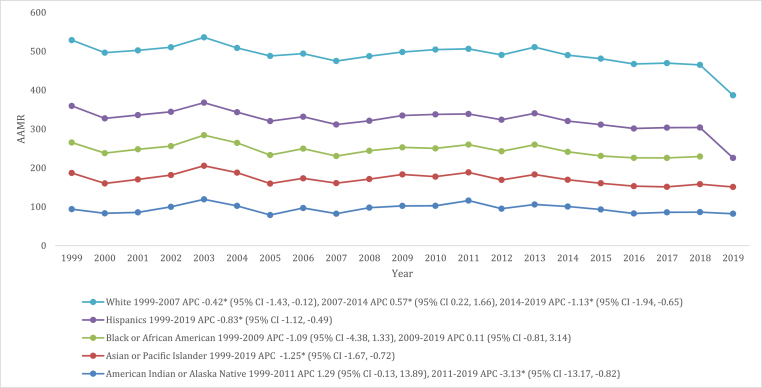
VHD-related mortality Trends in older adult population stratified for differential racial groups in 1999–2019.

In brief, the AAMRs for American Indian or Alaska Native populations remained stable from 1999 to 2011 (APC: 1.29 [95 % CI: 0.13 to 13.89]). This was followed by a steep decline from 2011 to 2019 (APC: 3.13 [95 % CI: 13.17 to −0.82]), while that of Asian or Pacific Islander and Hispanic or Latinos populations significantly declined between 1999 and 2019 (Asian or Pacific Islander: APC: 1.25 [95 % CI: 1.67 to −0.72]; Hispanic or Latinos: APC: 0.83 [95 % CI: 1.12 to −0.49]). With respect to Black or African American populations, AAMRs remained stable from 1999 to 2009 (APC: 1.09 [95 % CI: 4.38 to 1.33]). Similarly, from 2009 to 2019, another period of stability was observed (APC: 0.11 [95 % CI: 0.81 to 3.41]). A steady decline in AAMRs was observed among white individuals from 1999 to 2007 (APC: 0.42 [95 % CI: 1.43 to −0.12]). This was followed by a slight increase until 2014 (APC: 0.57 [95 % CI: 0.22 to 1.66]), after which another significant decline was noted from 2014 to 2019 (APC: 1.13 [95 % CI: 1.94 to −0.65]) (Fig. 2, Supplementary Table 3).

### 3.4 VHD-related AAMR stratified by geographic region

**Fig. 3:**
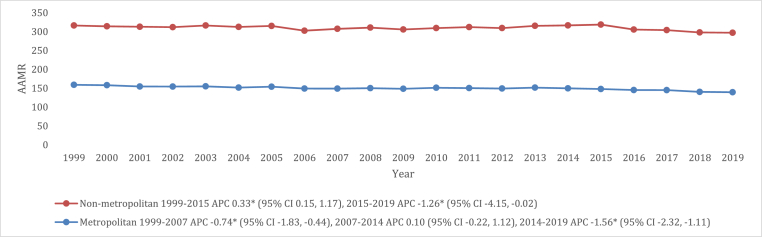
VHD-related mortality trends in older adult population residing in metropolitan vs. non-metropolitan areas in 1999–2019.

In a consistent pattern through the majority of the study period, nonmetropolitan areas manifested higher AAMRs for deaths related to VHD than metropolitan areas, with overall AAMRs of 160.5 (95 % CI: 159.6–161.3) and 149.5 (95 % CI: 149.1–149.9), respectively. Furthermore, the AAMRs of nonmetropolitan areas increased from 1999 to 2015 (APC: 0.33 [95 % CI: 0.15 to 1.17]), which was then followed by a steady decline from 2015 to 2019 (APC: 1.26 [95 % CI: 4.15 to −0.02]). On the contrary, AAMRs of metropolitan areas exhibited a steady decline from 1999 to 2007 (APC: 0.74 [95 % CI: 1.83 to −0.44]). This was followed by a period of stability until 2014 (APC: 0.10 [95 % CI: 0.22 to 1.12]), and finally, from 2014 to 2019, another significant decline in AAMRs was observed (APC: 1.56 [95 % CI: 2.32 to −1.11]) (Fig. 3, and Supplementary Table 4).

**Fig. 4:**
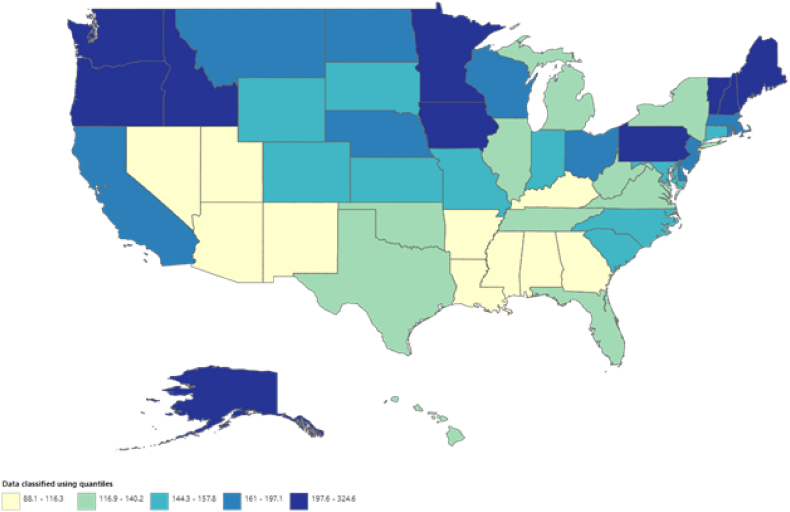
VHD-related mortality trends in older adult population stratified across 50 States in 1999–2019.

A significant disparity in VHD-related AAMRs has been observed on the state level, with the figures spanning from 88.1 (95 % CI: 85.1–91.1) in Mississippi to 324.6 (95 % CI [313.4, 335.8]) in Vermont. It is important to highlight that the states ranked in the top 90th percentile for AAMRs namely, Iowa, Alaska, Idaho, Pennsylvania, Minnesota, Maine, New Hampshire, Washington, Oregon, and Vermont, had approximately more than double the AAMRs compared with states that are ranked in the lower 10th percentile, namely, Arkansas, New Mexico, Louisiana, District of Columbia, Nevada, Arizona, Kentucky, Utah **(see** Fig. 4**, and** Supplementary Table 5**).**

### 3.5 VHD-related AAMR stratified by category of VHD

**Fig. 5:**
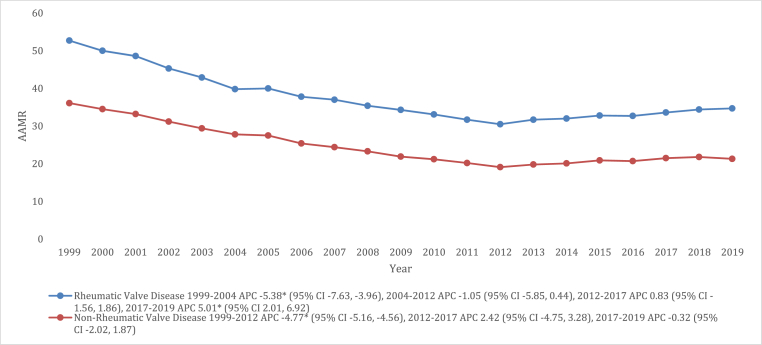
Valvular Heart Disease-related mortality Trends in older adult population for rheumatic vs. non-rheumatic valvular diseases in 1999–2019.

Throughout most of the study period, deaths related to VHDs were more frequent in non-rheumatic VHDs compared to rheumatic VHDs, with overall AAMRs of 24.3 (95 % CI: 24.1–24.4) and 12.9 (95 % CI: 12.8–13.0), respectively. Moreover, the AAMRs of non-rheumatic VHDs declined from 1999 to 2012 (APC: 4.77 [95 % CI: 5.16 to −4.56]), followed by periods of stability from 2012 to 2017 (APC: 2.42 [95 % CI: 4.75 to 3.28]) and 2017 to 2019 (APC: 0.32 [95 % CI: 2.02 to 1.87]). Similarly, AAMRs for rheumatic VHDs experienced a significant decline from 1999 to 2004 (APC: 5.38 [95 % CI: 7.63 to −3.96]), followed by stable rates from 2004 to 2012 (APC: 1.05 [95 % CI: 5.85 to 0.44]) and from 2012 to 2017 (APC: 0.83 [95 % CI: 1.56 to 1.86]). However, from 2017 to 2019, there was a notable increase in AAMRs for rheumatic VHDs (APC: 5.01 [95 % CI: 2.01 to 6.92]) (Fig. 5, and Supplementary Table 6).

**Fig. 6:**
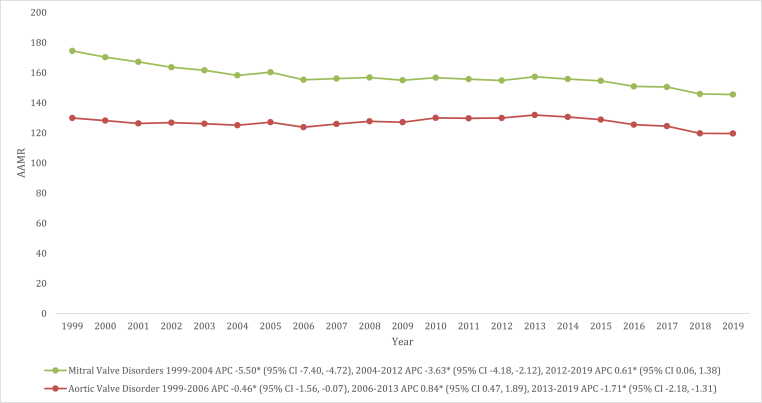
Valvular Heart Disease-related mortality Trends in older adult population for mitral vs. aortic valvular diseases in 1999–2019.

Aortic valve disorders (AVDs) have been the leading cause of VHD-related deaths throughout the study period in a consistent pattern as compared to mitral valve disorders (MVDs) with overall AAMRs of 126.7 (95 % CI: 126.3–127.0) and 30.0 (95 % CI: 29.8–30.1), respectively. The AAMRs of AVDs decreased from 1999 to 2006 (APC: 0.46 [95 % CI: 1.56 to −0.07]), followed by a steady incline from 2006 to 2013 (APC: 0.84 [95 % CI: 0.47 to 1.89]), and then a steep decline from 2013 to 2019 (APC: 1.71 [95 % CI: 2.18 to −1.31]). Similarly, AAMRs of MVDs exhibited a significant decline from 1999 to 2004 (APC: 5.50 [95 % CI: 7.40 to −4.72]) and 2004 to 2012 (APC: 3.63 [95 % CI: 4.18 to −2.12]). However, from 2012 to 2019, a significant incline in AAMRs was observed for MVDs (APC: 0.61 [95 % CI: 0.06 to 1.38]) (Fig. 6, and Supplementary Table 7).

### 3.6 VHD-related AAMR stratified by age

**Fig. 7:**
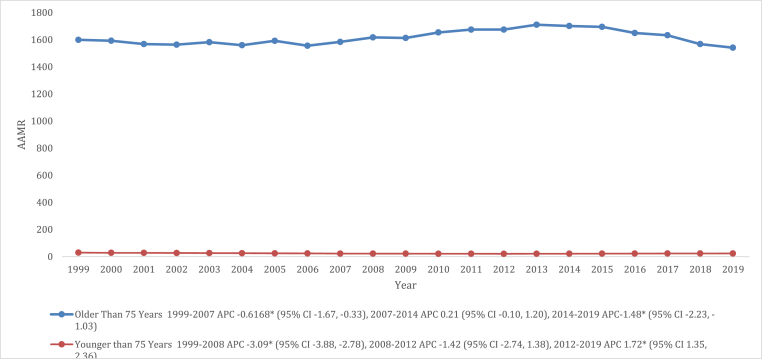
Valvular Heart Disease-related mortality Trends for Different Age Groups in 1999–2019.

In a consistent pattern, individuals aged 75 years and older manifested higher crude mortality rates for deaths related to VHD compared to those younger than 75 years, with an overall crude mortality rate of 1515.0 (95 % CI: 1511.4–1518.7) and 23.8 (95 % CI: 23.7–23.9), respectively. Furthermore, the crude death rate of the older age group decreased from 1999 to 2007 (APC: 0.61 [95 % CI: 1.67 to −0.33]), followed by a period of stability from 2007 to 2014 (APC: 0.21 [95 % CI: 0.10 to 1.20]) and then another significant decline from 2014 to 2019 (APC: 1.48 [95 % CI: 2.23 to −1.03]). In contrast, crude mortality rate of the younger age group exhibited a steep decline from 1999 to 2008 (APC: 3.09 [95 % CI: 3.88 to −2.78]), followed by a period of stability until 2012 (APC: 1.41 [95 % CI: 2.74 to 1.38]) and finally, from 2012 to 2019, a significant increase in crude death rate was noted (APC: 1.72 [95 % CI: 1.35 to 2.36]) (Fig. 7, and Supplementary Table 8).

## 4 Discussion

VHD poses a significant global public health challenge. Many parts of the world are still dealing with the negative outcomes of untreated rheumatic heart disease, a condition that can be largely prevented through prompt diagnosis and treatment. Additionally, middle- and high-income countries are witnessing an increase in the prevalence of calcific aortic and mitral diseases, partly due to an aging population. This public health issue is further complicated by high rates of infective endocarditis (IE), which results in substantial morbidity and mortality. Notably, research on VHD has not given adequate attention to considerations of gender, race, ethnicity, and geographical variations [11].

In this extensive 20-year analysis of nationwide mortality data obtained from the CDC, we have uncovered several noteworthy findings. Firstly, there was an initial period of decline in mortality rates related to VHD from 1999 to 2007, followed by a period of stability until 2014, and subsequently, a notable reduction in mortality from 2014 to 2019. Secondly, males exhibited higher VHD-related mortality rates compared to females consistently throughout the study. Thirdly, among the various racial and ethnic groups examined in this research, White adults displayed the highest AAMR for VHD. Fourthly, significant variations were observed between different regions, with states in the top 90th percentile, such as Pennsylvania, New Hampshire, Washington, Oregon, and Vermont, reporting AAMRs more than twice those of states in the lower 10th percentile, including Mississippi, Georgia, Alabama, Arkansas, and New Mexico. Additionally, non-metropolitan areas consistently exhibited higher mortality rates compared to metropolitan areas. Moreover, increased mortality rates were observed for non-rheumatic VHDs and AVDs when compared to rheumatic and MVDS, respectively. Finally, we also observed lower mortality rates associated with VHDs in the younger population (<75 years) when compared to their older counterparts (≥75 years). These findings hold substantial implications for public health policy.

The decrease in VHD-related deaths aligns with the progress made in assessing, identifying, and managing this population over the past twenty years. Notably, advancements such as enhanced genetic screening and risk factors stratification**,** including personal and family history, along with Artificial Intelligence**-**enhanced ambulatory electrocardiographic monitoring, echocardiography, computed tomography, and cardiac magnetic resonance imaging, have led to improved diagnostics and risk assessment [[12], [13], [14], [15]]. Consequently, VHD is being diagnosed more frequently but with reduced clinical severity in older adults, likely due to better clinical recognition [16].

Furthermore, therapeutic progress in VHD management, including newer approaches for percutaneous and surgical valve repair and implantation procedures, has broadened their applicability to a larger patient population, resulting in enhanced long-term survival [17,18]. Studies indicate a significant increase, approximately 5-fold for transcatheter aortic valve replacements and 10-fold for mitral valve replacements, in the past five years [19]. Additionally, early intervention, even among asymptomatic patients with severe VHD, has further reduced mortality rates and emphasized the need for novel population-based screening approaches [20]. However, it is crucial to note that despite these advancements, disparities in VHD mortality persist among specific demographic groups.

Our study findings indicate that mortality rates related to VHD remained stable or slightly increased until 2014 among older women and men, respectively, after which a consistent decline was observed. However, males consistently exhibited a higher mortality rate associated with VHD compared to females throughout this timeframe. This discrepancy can be attributed to a greater prevalence of concurrent risk factors in males, such as coronary artery disease (CAD), diabetes, hypertension (HTN), and smoking [21]. Additionally, delayed diagnosis also leads to females being under-referred for surgical or transcatheter valve repair procedures [22,23]. Interestingly, females, despite having worse short-term postoperative outcomes compared to men, tend to exhibit equal or even better long-term survival after treatment procedures, largely due to their longer life expectancy [24,25]. All these factors contribute to the higher prevalence of VHD in males and perpetuate the gender-based differences in VHD awareness and screening.

Furthermore, white patients with VHD had a higher mortality rate compared to other racial groups. This trend persisted despite an overall decline in mortality across different races throughout the study period. Although some studies have reported a lower prevalence of aortic stenosis in patients from racial and ethnic minorities compared to white patients, the differences in manifestation and presentation of aortic stenosis do not fully explain the variation in mortality [26]. Historically, racial and ethnic minorities have faced reduced access to diagnostic imaging and cardiac procedures, which may explain the disparities in prevalence based on hospital data [27,28]. These disparities in access could be attributed to financial constraints and potential implicit biases affecting referrals for VHD diagnosis and treatment. Additionally, the ability of primary care providers to identify which patients would benefit from screening echocardiography is low, as evidenced by a study from Brazil [29].

Significant healthcare disparities exist among racial and ethnic groups when it comes to the utilization of VHD surgical and transcatheter treatments, along with other structural heart interventions [28,30]. The reasons for the low utilization among racial and ethnic minorities are likely multifaceted, involving factors at the patient, provider, and healthcare system levels [31]. Ineffective patient communication and a lack of racial concordance may contribute to this issue, underscoring the importance of having a diverse physician workforce [32]. Thus, the underdiagnosis and lower referral rates among racial and ethnic minorities not only lead to underutilization of treatment procedures within those communities but also contribute to elevated VHD prevalence and, hence, mortality rates among the white population [33]. Addressing these disparities within institutions and implementing culturally tailored, multidisciplinary interventions may help mitigate the racial disparities observed in VHD-related mortality among adults.

In metropolitan regions, the AAMR linked to valvular heart disease has decreased over the last two decades. Conversely, there was a rise in VHD-related AAMR in non-metropolitan areas until 2015, but this was succeeded by a consistent decrease in recent years. Interestingly, nonmetropolitan areas have consistently maintained a higher AAMR than their metropolitan counterparts throughout the study period. This disparity can be attributed to several factors, including lower socioeconomic status in rural regions and a shortage of primary care physicians and cardiologists. Between 2002 and 2015, the number of primary care physicians declined at a rate twice as high in nonmetropolitan areas compared to urban areas [34]. The closure of a significant number of hospitals over the past decade further compounds these disparities. These regional differences may also be influenced by inconsistencies in outpatient cardiology practices, such as variations in the implementation of guideline-directed therapies, the impact of state regulations on Medicaid, restricted access to quality healthcare, a higher burden of comorbidities, and a prevalence of sedentary lifestyles [35]. These findings emphasize the need for extensive population-based studies in these regions to identify the primary factors contributing to these disparities.

One potential solution to address these issues is the establishment of a cloud-based image repository that utilizes standardized tools for communication between big academic setups and referring centers. This approach can expedite physician decision-making, enhance communication, and reduce patients' travel burden. Additionally, the implementation of a robust e-consult platform can provide comprehensive care for patients with VHD, covering consultations and follow-up care, thus addressing concerns about fragmented care [36].

Globally, rheumatic heart disease (RHD) is the most common valve pathology, whereas in developed countries like the US, degenerative non-rheumatic VHD prevails [37]. Our findings show that mortality rates related to VHD either remained stable or decreased during most of the study period for both non-rheumatic and rheumatic VHDs. However, our analysis revealed higher mortality rates from non-rheumatic VHDs compared to the rheumatic group in the US. Several factors could underlie this observation. Firstly, improved healthcare and longer life expectancy have led to an aging population with an increased incidence of degenerative valve diseases such as calcific aortic valve stenosis and degenerative mitral valve regurgitation [38]. Secondly, lifestyle changes have raised the prevalence of risk factors like hypertension, hyperlipidemia, diabetes, and smoking, contributing to the development and progression of non-rheumatic VHDs [38]. Thirdly, advancements in diagnostic methods like echocardiography enable earlier detection and diagnosis of non-rheumatic VHDs, resulting in higher reported mortality rates [[12], [13], [14], [15], [16]]. Moreover, the decline in rheumatic fever (RF) due to better healthcare practices and widespread antibiotic use has reduced the incidence of rheumatic VHDs [37]. Additionally, studies suggest a higher prevalence of coronary artery disease (CAD) in non-rheumatic VHDs, further increasing mortality rates in this group [38]. These factors collectively underscore the epidemiological shift towards non-rheumatic VHDs as a major cause of mortality in the US.

Despite an overall decline in mortality rates associated with aortic and mitral valve disorders over the past two decades, our analysis has indicated that deaths related to AVDs in the US exceed those associated with MVDs. Several factors could account for this difference. Firstly, AVD, especially aortic stenosis (AS), has a higher incidence and prevalence compared to MVD, primarily because they often result from age-related degenerative changes [38]. For instance, the prevalence of AS increases significantly with age, estimated at 0.2 % before the age of 65 and 2.8 % after 75 [39]. Conversely, MVD usually has multiple etiologies, including degenerative changes, RF, and congenital abnormalities, with RHD being a major contributor to mitral stenosis in older patients [40]. Secondly, well-established risk factors for aortic valve disorders, such as hypercholesterolemia, hypertension, and diabetes, are common comorbidities among the aging population in the US, predisposing individuals to the development and faster progression of AS [40]. Additionally, AVD is associated with potentially life-threatening complications like aortic dissection and left ventricular dysfunction, which may contribute to higher mortality rates. Nonetheless, in recent years, timely interventions and advancements in surgical and percutaneous valve repair procedures might account for decreased mortality rates associated with both aortic and mitral valve disorders [[17], [18], [19], [20]].

Similarly, our study showed a general decrease in mortality rates linked to VHD across both age categories. Additionally, our findings revealed higher VHD-related AAMRs in the older age bracket (≥75 years) compared to the younger group (<75 years) consistently throughout the study period. This can be attributed to the natural aging process. As individuals age, they are more likely to develop degenerative changes in the heart valves, such as calcification and fibrosis, leading to the development of VHD [38]. Furthermore, older individuals often have comorbidities such as hypertension, diabetes, and CAD, which further increase their risk of developing VHD and experiencing adverse outcomes [41]. However, the advancements in medical treatment, including the use of medications like statins, antiplatelet agents, and antihypertensives, and improved management of associated risk factors through public health initiatives like healthy eating campaigns, smoking cessation, and exercise programs, have led to better disease control, and patient outcomes [16,20]. These combined efforts might account for the notable decline in mortality associated with VHD in recent years, especially in the younger population.

### 4.1 Limitations

This study has several limitations that warrant consideration. Firstly, the use of International Statistical Classification of Diseases codes (ICD-10) and reliance on death certificates introduces the potential for errors in classifying or omitting VHD as a cause of death [42]. Moreover, IE was not specifically evaluated in our analysis as a cause of death. These discrepancies in diagnosis and disease classification can impact mortality data. Secondly, we employed Age-Adjusted Mortality Rates (AAMRs) per 1,000,000 individuals to analyze deaths attributed to VHD across distinct age brackets (≥75 years vs. < 75 years) due to insufficient data from the CDC regarding AAMRs per 100,000 individuals. Thirdly, the database lacks essential clinical variables that could provide a more comprehensive description of disease characteristics. These variables include details about the specific VHD phenotype, vital signs, laboratory results, ejection fraction, genetic analysis, and imaging data, which are critical for a thorough understanding of the disease phenotype, its severity, and the presence of factors that affect prognosis. Additionally, the rise in electronic health record diagnosis of VHD may lead to increased reporting of VHD-related deaths on death certificates. Furthermore, the database does not contain information on the treatments received by individuals, such as medical therapies, valvular repair, and implantation by surgical or percutaneous approaches. Lastly, it lacks data related to social and economic determinants of health, which can significantly impact access to healthcare and, subsequently, influence mortality rates among different demographic groups.

## 5 Conclusion

Our study revealed a decline in VHD mortality in the United States from 1999 to 2019. Nevertheless, disparities persist, particularly concerning gender, race, age, geographic regions, and the type of VHD. Notably, White adults, males, and older adults (≥75 years), nonmetropolitan areas, non-rheumatic VHD, and AVDS exhibited the highest age-adjusted mortality rates. Addressing these disparities requires targeted health policies aimed at reducing VHD mortality equitably, emphasizing prevention, early diagnosis, and prompt referrals.

## Funding

This research did not receive any specific grant from funding agencies in the public, commercial, or not-for-profit sectors.

## CRediT authorship contribution statement

**Eman Ali:** Conceptualization, Data curation, Formal analysis. **Yusra Mashkoor:** Data curation, Formal analysis, Writing – original draft. **Fakhar Latif:** Data curation, Resources, Software. **Fnu Zafrullah:** Methodology, Software, Writing – original draft. **Waleed Alruwaili:** Data curation, Formal analysis, Resources. **Sameh Nassar:** Software, Visualization, Writing – original draft. **Karthik Gonuguntla:** Methodology, Validation, Writing – original draft. **Harshith Thyagaturu:** Data curation, Investigation, Writing – original draft. **Mohammad Kawsara:** Supervision, Validation, Visualization, Writing – review & editing. **Ramesh Daggubati:** Data curation, Software, Writing – review & editing. **Yasar Sattar:** Conceptualization, Project administration, Writing – review & editing. **Muhammad Sohaib Asghar:** Formal analysis, Supervision, Writing – review & editing.

## Declaration of competing interest

None of the authors have any financial or industrial conflict of interest.
