# Supplementary material for: Demographics and mortality trends of valvular heart disease in older adults in the United States: Insights from CDC-wonder database 1999–2019
Source: Int J Cardiol Cardiovasc Risk Prev. 2024 Aug 17;22:200321. doi: 10.1016/j.ijcrp.2024.200321 (PMC11380170; doi:10.1016/j.ijcrp.2024.200321)
Supplement: Multimedia component 1 [file mmc1.docx]

Supplementary Tables

Table 1: Overall AAMRS and 95% Confidence Intervals from 1999-2019.

| **Year** | **Age Adjusted Rate per 100,000** | **Age Adjusted Rate Lower 95% Confidence Interval** | **Age Adjusted Rate Upper 95% Confidence Interval** |
| --- | --- | --- | --- |
| 1999 | 158.8 | 156.9 | 160.8 |
| 2000 | 157.8 | 155.9 | 159.7 |
| 2001 | 155.4 | 153.5 | 157.3 |
| 2002 | 155 | 153.2 | 156.9 |
| 2003 | 156.3 | 154.4 | 158.2 |
| 2004 | 153.6 | 151.7 | 155.4 |
| 2005 | 155.4 | 153.6 | 157.2 |
| 2006 | 150 | 148.3 | 151.8 |
| 2007 | 150.8 | 149.1 | 152.6 |
| 2008 | 152.2 | 150.5 | 154 |
| 2009 | 150.1 | 148.4 | 151.8 |
| 2010 | 152.5 | 150.8 | 154.2 |
| 2011 | 152.4 | 150.7 | 154.1 |
| 2012 | 151.4 | 149.7 | 153 |
| 2013 | 153.9 | 152.2 | 155.6 |
| 2014 | 152.7 | 151.1 | 154.4 |
| 2015 | 152 | 150.4 | 153.6 |
| 2016 | 147.9 | 146.3 | 149.5 |
| 2017 | 147.5 | 146 | 149.1 |
| 2018 | 143.4 | 141.9 | 144.9 |
| 2019 | 142.6 | 141 | 144.1 |

Table 2: AAMRs and 95% Confidence Intervals stratified for gender from 1999-2019.

| **Gender** | **Year** | **Age Adjusted Rate per 100,000** | **Age Adjusted Rate Lower 95% Confidence Interval** | **Age Adjusted Rate Upper 95% Confidence Interval** |
| --- | --- | --- | --- | --- |
| Female | 1999 | 146.8 | 144.5 | 149.1 |
| Female | 2000 | 146.3 | 144.1 | 148.6 |
| Female | 2001 | 144.2 | 142 | 146.4 |
| Female | 2002 | 143.4 | 141.2 | 145.6 |
| Female | 2003 | 144.1 | 141.9 | 146.3 |
| Female | 2004 | 141.5 | 139.3 | 143.7 |
| Female | 2005 | 145 | 142.8 | 147.1 |
| Female | 2006 | 139.2 | 137.1 | 141.4 |
| Female | 2007 | 138 | 135.9 | 140.1 |
| Female | 2008 | 139.1 | 137 | 141.2 |
| Female | 2009 | 138.4 | 136.3 | 140.4 |
| Female | 2010 | 138.3 | 136.2 | 140.3 |
| Female | 2011 | 139.1 | 137 | 141.1 |
| Female | 2012 | 135.6 | 133.6 | 137.6 |
| Female | 2013 | 139.2 | 137.2 | 141.3 |
| Female | 2014 | 137.2 | 135.2 | 139.2 |
| Female | 2015 | 137.3 | 135.3 | 139.2 |
| Female | 2016 | 133 | 131.1 | 135 |
| Female | 2017 | 133 | 131.1 | 134.9 |
| Female | 2018 | 130.3 | 128.5 | 132.2 |
| Female | 2019 | 128.7 | 126.8 | 130.5 |
| Male | 1999 | 180.1 | 176.5 | 183.7 |
| Male | 2000 | 178.6 | 175.1 | 182.2 |
| Male | 2001 | 174.8 | 171.3 | 178.3 |
| Male | 2002 | 175.7 | 172.3 | 179.2 |
| Male | 2003 | 177.8 | 174.4 | 181.2 |
| Male | 2004 | 174.9 | 171.5 | 178.2 |
| Male | 2005 | 173.7 | 170.4 | 177 |
| Male | 2006 | 168.8 | 165.6 | 172 |
| Male | 2007 | 172.9 | 169.7 | 176 |
| Male | 2008 | 175.1 | 171.9 | 178.2 |
| Male | 2009 | 170.1 | 167.1 | 173.2 |
| Male | 2010 | 176.3 | 173.3 | 179.4 |
| Male | 2011 | 174.6 | 171.6 | 177.6 |
| Male | 2012 | 177.3 | 174.3 | 180.3 |
| Male | 2013 | 178 | 175 | 180.9 |
| Male | 2014 | 177.5 | 174.6 | 180.4 |
| Male | 2015 | 174.8 | 171.9 | 177.6 |
| Male | 2016 | 171.2 | 168.4 | 174 |
| Male | 2017 | 169.8 | 167.1 | 172.6 |
| Male | 2018 | 163.3 | 160.7 | 166 |
| Male | 2019 | 163.4 | 160.8 | 166 |

Table 3: AAMRs and 95% Confidence Intervals stratified for race from 1999-2019

| **Race** | **Year** | **Age Adjusted Rate per 100,000** | **Age Adjusted Rate Lower 95% Confidence Interval** | **Age Adjusted Rate Upper 95% Confidence Interval** |
| --- | --- | --- | --- | --- |
| American Indian or Alaska Native | 1999 | 93.9 | 68.3 | 126.1 |
| American Indian or Alaska Native | 2000 | 83.2 | 60.4 | 111.6 |
| American Indian or Alaska Native | 2001 | 85.6 | 62.4 | 114.5 |
| American Indian or Alaska Native | 2002 | 99.8 | 75 | 130.2 |
| American Indian or Alaska Native | 2003 | 119.1 | 91.7 | 152.1 |
| American Indian or Alaska Native | 2004 | 102.3 | 77.5 | 132.6 |
| American Indian or Alaska Native | 2005 | 78.8 | 57.5 | 105.4 |
| American Indian or Alaska Native | 2006 | 96.8 | 73.3 | 125.4 |
| American Indian or Alaska Native | 2007 | 82.2 | 61.2 | 108.1 |
| American Indian or Alaska Native | 2008 | 97.7 | 74.9 | 125.3 |
| American Indian or Alaska Native | 2009 | 102.3 | 79.5 | 129.7 |
| American Indian or Alaska Native | 2010 | 102.5 | 79.8 | 129.7 |
| American Indian or Alaska Native | 2011 | 115.9 | 92.7 | 143.2 |
| American Indian or Alaska Native | 2012 | 95.1 | 74.8 | 119.2 |
| American Indian or Alaska Native | 2013 | 105.9 | 84.9 | 130.4 |
| American Indian or Alaska Native | 2014 | 100.7 | 80.9 | 123.9 |
| American Indian or Alaska Native | 2015 | 93 | 74.6 | 114.6 |
| American Indian or Alaska Native | 2016 | 82.8 | 66 | 102.7 |
| American Indian or Alaska Native | 2017 | 85.7 | 69 | 105.3 |
| American Indian or Alaska Native | 2018 | 86.3 | 70.1 | 105.2 |
| American Indian or Alaska Native | 2019 | 82.1 | 66.7 | 100.1 |
| Asian or Pacific Islander | 1999 | 93 | 81.6 | 104.5 |
| Asian or Pacific Islander | 2000 | 76.9 | 66.8 | 87.1 |
| Asian or Pacific Islander | 2001 | 84.7 | 74.6 | 94.7 |
| Asian or Pacific Islander | 2002 | 81.7 | 72.1 | 91.2 |
| Asian or Pacific Islander | 2003 | 86.4 | 76.9 | 95.8 |
| Asian or Pacific Islander | 2004 | 85.5 | 76.4 | 94.6 |
| Asian or Pacific Islander | 2005 | 80.9 | 72.3 | 89.5 |
| Asian or Pacific Islander | 2006 | 76.4 | 68.3 | 84.5 |
| Asian or Pacific Islander | 2007 | 78.6 | 70.6 | 86.5 |
| Asian or Pacific Islander | 2008 | 73.6 | 66.2 | 81 |
| Asian or Pacific Islander | 2009 | 80.9 | 73.4 | 88.4 |
| Asian or Pacific Islander | 2010 | 75.1 | 68 | 82.1 |
| Asian or Pacific Islander | 2011 | 72.5 | 65.9 | 79.1 |
| Asian or Pacific Islander | 2012 | 74 | 67.6 | 80.4 |
| Asian or Pacific Islander | 2013 | 77.1 | 70.8 | 83.4 |
| Asian or Pacific Islander | 2014 | 68.8 | 63.1 | 74.6 |
| Asian or Pacific Islander | 2015 | 67.5 | 62.1 | 73 |
| Asian or Pacific Islander | 2016 | 70.2 | 64.8 | 75.6 |
| Asian or Pacific Islander | 2017 | 65.5 | 60.5 | 70.6 |
| Asian or Pacific Islander | 2018 | 71.8 | 66.7 | 77 |
| Asian or Pacific Islander | 2019 | 68.9 | 64 | 73.8 |
| Black or African American | 1999 | 81.8 | 76.7 | 86.9 |
| Black or African American | 2000 | 78.5 | 73.5 | 83.4 |
| Black or African American | 2001 | 77.9 | 72.9 | 82.8 |
| Black or African American | 2002 | 77.5 | 72.5 | 82.4 |
| Black or African American | 2003 | 74.6 | 69.8 | 79.4 |
| Black or African American | 2004 | 78.7 | 73.8 | 83.6 |
| Black or African American | 2005 | 76.6 | 71.9 | 81.4 |
| Black or African American | 2006 | 73.3 | 68.7 | 77.9 |
| Black or African American | 2007 | 76.3 | 71.6 | 80.9 |
| Black or African American | 2008 | 69.7 | 65.3 | 74.1 |
| Black or African American | 2009 | 72.7 | 68.3 | 77.2 |
| Black or African American | 2010 | 69.6 | 65.3 | 73.9 |
| Black or African American | 2011 | 72.7 | 68.4 | 77 |
| Black or African American | 2012 | 71.5 | 67.3 | 75.7 |
| Black or African American | 2013 | 73.7 | 69.5 | 77.9 |
| Black or African American | 2014 | 76.8 | 72.5 | 81 |
| Black or African American | 2015 | 71.8 | 67.8 | 75.9 |
| Black or African American | 2016 | 70.3 | 66.4 | 74.2 |
| Black or African American | 2017 | 72.9 | 69 | 76.8 |
| Black or African American | 2018 | 74.5 | 70.6 | 78.4 |
| Black or African American | 2019 | 71.2 | 67.4 | 74.9 |
| Hispanic or Latino | 1999 | 94.1 | 86.3 | 101.9 |
| Hispanic or Latino | 2000 | 89.4 | 82 | 96.8 |
| Hispanic or Latino | 2001 | 88.1 | 81 | 95.2 |
| Hispanic or Latino | 2002 | 88.2 | 81.3 | 95.2 |
| Hispanic or Latino | 2003 | 83.6 | 77 | 90.2 |
| Hispanic or Latino | 2004 | 78.9 | 72.7 | 85.1 |
| Hispanic or Latino | 2005 | 87.3 | 81 | 93.7 |
| Hispanic or Latino | 2006 | 82.1 | 76.1 | 88.1 |
| Hispanic or Latino | 2007 | 81.1 | 75.3 | 86.9 |
| Hispanic or Latino | 2008 | 77.2 | 71.7 | 82.7 |
| Hispanic or Latino | 2009 | 81.9 | 76.4 | 87.3 |
| Hispanic or Latino | 2010 | 87.4 | 81.9 | 93 |
| Hispanic or Latino | 2011 | 78.9 | 73.9 | 84 |
| Hispanic or Latino | 2012 | 81.1 | 76.2 | 86.1 |
| Hispanic or Latino | 2013 | 80.6 | 75.8 | 85.3 |
| Hispanic or Latino | 2014 | 79.2 | 74.6 | 83.8 |
| Hispanic or Latino | 2015 | 80.5 | 76 | 84.9 |
| Hispanic or Latino | 2016 | 75.5 | 71.3 | 79.7 |
| Hispanic or Latino | 2017 | 77.9 | 73.7 | 82.1 |
| Hispanic or Latino | 2018 | 74.6 | 70.6 | 78.6 |
| Hispanic or Latino | 2019 | 74.6 | 70.7 | 78.5 |
| White | 1999 | 169.2 | 167.1 | 171.4 |
| White | 2000 | 169 | 166.9 | 171.2 |
| White | 2001 | 166.4 | 164.3 | 168.5 |
| White | 2002 | 166.1 | 164.1 | 168.2 |
| White | 2003 | 168.1 | 166.1 | 170.2 |
| White | 2004 | 165.2 | 163.2 | 167.2 |
| White | 2005 | 167.7 | 165.6 | 169.7 |
| White | 2006 | 162.4 | 160.4 | 164.4 |
| White | 2007 | 163.4 | 161.5 | 165.4 |
| White | 2008 | 166.2 | 164.2 | 168.2 |
| White | 2009 | 163.4 | 161.5 | 165.4 |
| White | 2010 | 166.7 | 164.8 | 168.7 |
| White | 2011 | 167.5 | 165.5 | 169.4 |
| White | 2012 | 166.6 | 164.7 | 168.5 |
| White | 2013 | 170.2 | 168.2 | 172.1 |
| White | 2014 | 169.6 | 167.7 | 171.6 |
| White | 2015 | 169.7 | 167.8 | 171.6 |
| White | 2016 | 165.9 | 164 | 167.8 |
| White | 2017 | 166 | 164.2 | 167.9 |
| White | 2018 | 161.1 | 159.3 | 162.9 |
| White | 2019 | 161.2 | 159.4 | 163 |

Table 4: AAMRs and 95% Confidence Intervals for Metropolitan vs. Non-metropolitan areas from 1999-2019.

| **Metropolitan vs. non-metropolitan area** | **Year** | **Age Adjusted Rate per 100,000** | **Age Adjusted Rate Lower 95% Confidence Interval** | **Age Adjusted Rate Upper 95% Confidence Interval** |
| --- | --- | --- | --- | --- |
| Metropolitan | 1999 | 159.2 | 157.1 | 161.4 |
| Metropolitan | 2000 | 158.2 | 156.1 | 160.4 |
| Metropolitan | 2001 | 154.7 | 152.6 | 156.8 |
| Metropolitan | 2002 | 154.5 | 152.4 | 156.5 |
| Metropolitan | 2003 | 155.1 | 153 | 157.1 |
| Metropolitan | 2004 | 151.8 | 149.8 | 153.9 |
| Metropolitan | 2005 | 154.1 | 152.1 | 156.1 |
| Metropolitan | 2006 | 149.3 | 147.3 | 151.3 |
| Metropolitan | 2007 | 149.1 | 147.1 | 151 |
| Metropolitan | 2008 | 150.3 | 148.4 | 152.3 |
| Metropolitan | 2009 | 148.6 | 146.7 | 150.5 |
| Metropolitan | 2010 | 151.3 | 149.4 | 153.2 |
| Metropolitan | 2011 | 150.4 | 148.5 | 152.2 |
| Metropolitan | 2012 | 149.4 | 147.6 | 151.3 |
| Metropolitan | 2013 | 151.7 | 149.9 | 153.5 |
| Metropolitan | 2014 | 149.8 | 148 | 151.6 |
| Metropolitan | 2015 | 148 | 146.2 | 149.8 |
| Metropolitan | 2016 | 145.3 | 143.6 | 147 |
| Metropolitan | 2017 | 145.1 | 143.4 | 146.8 |
| Metropolitan | 2018 | 140.4 | 138.8 | 142.1 |
| Metropolitan | 2019 | 139.5 | 137.9 | 141.1 |
| Non-metropolitan | 1999 | 157.3 | 153 | 161.7 |
| Non-metropolitan | 2000 | 156.1 | 151.8 | 160.4 |
| Non-metropolitan | 2001 | 158.5 | 154.2 | 162.8 |
| Non-metropolitan | 2002 | 157.6 | 153.3 | 161.9 |
| Non-metropolitan | 2003 | 161.4 | 157.1 | 165.7 |
| Non-metropolitan | 2004 | 160.9 | 156.6 | 165.3 |
| Non-metropolitan | 2005 | 161.2 | 156.9 | 165.5 |
| Non-metropolitan | 2006 | 153.4 | 149.2 | 157.6 |
| Non-metropolitan | 2007 | 158.6 | 154.4 | 162.8 |
| Non-metropolitan | 2008 | 160.6 | 156.4 | 164.8 |
| Non-metropolitan | 2009 | 157.3 | 153.1 | 161.4 |
| Non-metropolitan | 2010 | 158.3 | 154.2 | 162.5 |
| Non-metropolitan | 2011 | 161.9 | 157.8 | 166.1 |
| Non-metropolitan | 2012 | 160.2 | 156.1 | 164.3 |
| Non-metropolitan | 2013 | 163.9 | 159.7 | 168 |
| Non-metropolitan | 2014 | 167 | 162.9 | 171.2 |
| Non-metropolitan | 2015 | 170.9 | 166.7 | 175 |
| Non-metropolitan | 2016 | 160.5 | 156.5 | 164.5 |
| Non-metropolitan | 2017 | 159.1 | 155.1 | 163 |
| Non-metropolitan | 2018 | 157.7 | 153.8 | 161.6 |
| Non-metropolitan | 2019 | 157.8 | 153.9 | 161.6 |

Table 5: Age adjusted mortality rates in older population with valvular heart disease in 50 States.

| **State** | **Age Adjusted Rate per 100,000** | **Age Adjusted Rate Lower 95% Confidence Interval** | **Age Adjusted Rate Upper 95% Confidence Interval** |
| --- | --- | --- | --- |
| Alabama | 94.8 | 92.4 | 97.1 |
| Alaska | 203.9 | 190.3 | 217.4 |
| Arizona | 110.8 | 108.6 | 113 |
| Arkansas | 98.4 | 95.5 | 101.4 |
| California | 168.3 | 167.1 | 169.4 |
| Colorado | 146.7 | 143.6 | 149.9 |
| Connecticut | 155.2 | 152.1 | 158.3 |
| Delaware | 179 | 171.7 | 186.3 |
| District of Columbia | 104.2 | 97.1 | 111.3 |
| Florida | 116.9 | 115.8 | 118 |
| Georgia | 91.2 | 89.3 | 93.1 |
| Hawaii | 137.8 | 133 | 142.6 |
| Idaho | 204.8 | 198.5 | 211.1 |
| Illinois | 133.9 | 132.2 | 135.6 |
| Indiana | 156 | 153.4 | 158.6 |
| Iowa | 197.6 | 193.9 | 201.3 |
| Kansas | 144.3 | 140.7 | 147.8 |
| Kentucky | 111 | 108.3 | 113.7 |
| Louisiana | 99.9 | 97.3 | 102.5 |
| Maine | 219 | 213 | 225.1 |
| Maryland | 156.5 | 153.7 | 159.3 |
| Massachusetts | 165.6 | 163.2 | 168 |
| Michigan | 140.2 | 138.3 | 142.1 |
| Minnesota | 219.1 | 215.9 | 222.4 |
| Mississippi | 88.1 | 85.1 | 91.1 |
| Missouri | 152.9 | 150.4 | 155.5 |
| Montana | 171.8 | 165.3 | 178.2 |
| Nebraska | 183.5 | 178.6 | 188.4 |
| Nevada | 109.9 | 106 | 113.8 |
| New Hampshire | 223.4 | 216.7 | 230 |
| New Jersey | 177.5 | 175.3 | 179.7 |
| New Mexico | 98.7 | 95 | 102.5 |
| New York | 121.6 | 120.4 | 122.8 |
| North Carolina | 148.1 | 146 | 150.3 |
| North Dakota | 161 | 153.9 | 168.1 |
| Ohio | 166 | 164.1 | 167.8 |
| Oklahoma | 117.1 | 114.2 | 120 |
| Oregon | 290.4 | 286.1 | 294.8 |
| Pennsylvania | 209.9 | 208 | 211.7 |
| Rhode Island | 169.3 | 163.4 | 175.1 |
| South Carolina | 147.7 | 144.7 | 150.8 |
| South Dakota | 153.7 | 147.2 | 160.1 |
| Tennessee | 137.6 | 135.1 | 140.1 |
| Texas | 118.5 | 117.2 | 119.8 |
| Utah | 116.3 | 112.1 | 120.6 |
| Vermont | 324.6 | 313.4 | 335.8 |
| Virginia | 134.8 | 132.6 | 137.1 |
| Washington | 258.5 | 255.2 | 261.8 |
| West Virginia | 136 | 131.8 | 140.1 |
| Wisconsin | 197.1 | 194.2 | 200 |
| Wyoming | 157.8 | 148.5 | 167 |

Table 6: Age adjusted mortality rates in older population with valvular heart disease in rheumatic vs non-rheumatic valvular disease from 1999-2019.

| **Type of Valvular Heart Disease** | **Year** | **Age Adjusted Rate per 100,000** | **Age Adjusted Rate Lower 95% Confidence Interval** | **Age Adjusted Rate Upper 95% Confidence Interval** |
| --- | --- | --- | --- | --- |
| Rheumatic Valve Disease | 1999 | 16.6 | 16 | 17.3 |
| Rheumatic Valve Disease | 2000 | 15.5 | 14.9 | 16.1 |
| Rheumatic Valve Disease | 2001 | 15.4 | 14.9 | 16 |
| Rheumatic Valve Disease | 2002 | 14.1 | 13.5 | 14.6 |
| Rheumatic Valve Disease | 2003 | 13.5 | 12.9 | 14 |
| Rheumatic Valve Disease | 2004 | 12 | 11.4 | 12.5 |
| Rheumatic Valve Disease | 2005 | 12.5 | 12 | 13 |
| Rheumatic Valve Disease | 2006 | 12.4 | 11.9 | 13 |
| Rheumatic Valve Disease | 2007 | 12.6 | 12.1 | 13.1 |
| Rheumatic Valve Disease | 2008 | 12.1 | 11.6 | 12.6 |
| Rheumatic Valve Disease | 2009 | 12.4 | 11.9 | 12.9 |
| Rheumatic Valve Disease | 2010 | 11.9 | 11.4 | 12.3 |
| Rheumatic Valve Disease | 2011 | 11.5 | 11 | 12 |
| Rheumatic Valve Disease | 2012 | 11.4 | 10.9 | 11.9 |
| Rheumatic Valve Disease | 2013 | 11.9 | 11.4 | 12.3 |
| Rheumatic Valve Disease | 2014 | 11.9 | 11.4 | 12.4 |
| Rheumatic Valve Disease | 2015 | 11.9 | 11.5 | 12.4 |
| Rheumatic Valve Disease | 2016 | 12 | 11.5 | 12.4 |
| Rheumatic Valve Disease | 2017 | 12.1 | 11.7 | 12.6 |
| Rheumatic Valve Disease | 2018 | 12.6 | 12.2 | 13.1 |
| Rheumatic Valve Disease | 2019 | 13.4 | 13 | 13.9 |
| Non-Rheumatic Valve Disease | 1999 | 36.1 | 35.2 | 37 |
| Non-Rheumatic Valve Disease | 2000 | 34.5 | 33.6 | 35.4 |
| Non-Rheumatic Valve Disease | 2001 | 33.2 | 32.3 | 34.1 |
| Non-Rheumatic Valve Disease | 2002 | 31.2 | 30.3 | 32 |
| Non-Rheumatic Valve Disease | 2003 | 29.4 | 28.6 | 30.2 |
| Non-Rheumatic Valve Disease | 2004 | 27.8 | 27 | 28.6 |
| Non-Rheumatic Valve Disease | 2005 | 27.5 | 26.7 | 28.3 |
| Non-Rheumatic Valve Disease | 2006 | 25.4 | 24.7 | 26.2 |
| Non-Rheumatic Valve Disease | 2007 | 24.4 | 23.7 | 25.1 |
| Non-Rheumatic Valve Disease | 2008 | 23.3 | 22.6 | 24 |
| Non-Rheumatic Valve Disease | 2009 | 21.9 | 21.2 | 22.5 |
| Non-Rheumatic Valve Disease | 2010 | 21.2 | 20.6 | 21.9 |
| Non-Rheumatic Valve Disease | 2011 | 20.2 | 19.5 | 20.8 |
| Non-Rheumatic Valve Disease | 2012 | 19.1 | 18.5 | 19.7 |
| Non-Rheumatic Valve Disease | 2013 | 19.8 | 19.1 | 20.4 |
| Non-Rheumatic Valve Disease | 2014 | 20.1 | 19.5 | 20.7 |
| Non-Rheumatic Valve Disease | 2015 | 20.9 | 20.3 | 21.5 |
| Non-Rheumatic Valve Disease | 2016 | 20.7 | 20.1 | 21.3 |
| Non-Rheumatic Valve Disease | 2017 | 21.5 | 20.9 | 22.1 |
| Non-Rheumatic Valve Disease | 2018 | 21.8 | 21.2 | 22.4 |
| Non-Rheumatic Valve Disease | 2019 | 21.3 | 20.7 | 21.8 |

Table 7: Age adjusted mortality rates in older population with valvular heart disease in aortic vs mitral valve disorders from 1999-2019.

| **Type of Valvular Heart Disease** | **Year** | **Age Adjusted Rate per 100,000** | **Age Adjusted Rate Lower 95% Confidence Interval** | **Age Adjusted Rate Upper 95% Confidence Interval** |
| --- | --- | --- | --- | --- |
| Mitral Valve Disorders | 1999 | 44.6 | 43.5 | 45.6 |
| Mitral Valve Disorders | 2000 | 42.1 | 41.1 | 43 |
| Mitral Valve Disorders | 2001 | 40.9 | 40 | 41.9 |
| Mitral Valve Disorders | 2002 | 36.8 | 35.9 | 37.7 |
| Mitral Valve Disorders | 2003 | 35.5 | 34.6 | 36.4 |
| Mitral Valve Disorders | 2004 | 33.1 | 32.2 | 33.9 |
| Mitral Valve Disorders | 2005 | 33.2 | 32.3 | 34 |
| Mitral Valve Disorders | 2006 | 31.5 | 30.7 | 32.3 |
| Mitral Valve Disorders | 2007 | 30.2 | 29.4 | 31 |
| Mitral Valve Disorders | 2008 | 29.1 | 28.3 | 29.9 |
| Mitral Valve Disorders | 2009 | 27.9 | 27.1 | 28.6 |
| Mitral Valve Disorders | 2010 | 26.7 | 26 | 27.5 |
| Mitral Valve Disorders | 2011 | 26 | 25.3 | 26.7 |
| Mitral Valve Disorders | 2012 | 24.9 | 24.2 | 25.6 |
| Mitral Valve Disorders | 2013 | 25.4 | 24.7 | 26.1 |
| Mitral Valve Disorders | 2014 | 25.2 | 24.5 | 25.9 |
| Mitral Valve Disorders | 2015 | 25.8 | 25.1 | 26.5 |
| Mitral Valve Disorders | 2016 | 25.4 | 24.7 | 26.1 |
| Mitral Valve Disorders | 2017 | 26 | 25.3 | 26.7 |
| Mitral Valve Disorders | 2018 | 26.2 | 25.5 | 26.8 |
| Mitral Valve Disorders | 2019 | 25.9 | 25.2 | 26.6 |
| Aortic Valve Disorder | 1999 | 129.9 | 128.1 | 131.6 |
| Aortic Valve Disorder | 2000 | 128.2 | 126.5 | 129.9 |
| Aortic Valve Disorder | 2001 | 126.3 | 124.6 | 128 |
| Aortic Valve Disorder | 2002 | 126.8 | 125.1 | 128.5 |
| Aortic Valve Disorder | 2003 | 126.1 | 124.4 | 127.8 |
| Aortic Valve Disorder | 2004 | 125.1 | 123.4 | 126.7 |
| Aortic Valve Disorder | 2005 | 127.1 | 125.4 | 128.7 |
| Aortic Valve Disorder | 2006 | 123.8 | 122.2 | 125.4 |
| Aortic Valve Disorder | 2007 | 125.9 | 124.2 | 127.5 |
| Aortic Valve Disorder | 2008 | 127.7 | 126.1 | 129.3 |
| Aortic Valve Disorder | 2009 | 127.1 | 125.5 | 128.7 |
| Aortic Valve Disorder | 2010 | 130 | 128.4 | 131.6 |
| Aortic Valve Disorder | 2011 | 129.7 | 128.1 | 131.3 |
| Aortic Valve Disorder | 2012 | 129.9 | 128.3 | 131.4 |
| Aortic Valve Disorder | 2013 | 131.9 | 130.3 | 133.4 |
| Aortic Valve Disorder | 2014 | 130.6 | 129 | 132.1 |
| Aortic Valve Disorder | 2015 | 128.8 | 127.3 | 130.3 |
| Aortic Valve Disorder | 2016 | 125.5 | 124.1 | 127 |
| Aortic Valve Disorder | 2017 | 124.5 | 123 | 125.9 |
| Aortic Valve Disorder | 2018 | 119.7 | 118.3 | 121.1 |
| Aortic Valve Disorder | 2019 | 119.6 | 118.2 | 121 |

Table 8: Age adjusted mortality rates in younger population with valvular heart disease from 1999-2019.

| **Age Group** | **Year** | **Age Adjusted Rate per**  **1,000,000** | **Age Adjusted Rate Lower 95% Confidence Interval** | **Age Adjusted Rate Upper 95% Confidence Interval** |
| --- | --- | --- | --- | --- |
| Younger than 75 Years | 1999 | 29.9 | 29.2 | 30.5 |
| Younger than 75 Years | 2000 | 28.3 | 27.7 | 29 |
| Younger than 75 Years | 2001 | 28 | 27.3 | 28.6 |
| Younger than 75 Years | 2002 | 27.2 | 26.6 | 27.8 |
| Younger than 75 Years | 2003 | 26.4 | 25.8 | 27 |
| Younger than 75 Years | 2004 | 25.7 | 25.1 | 26.3 |
| Younger than 75 Years | 2005 | 24.7 | 24.1 | 25.2 |
| Younger than 75 Years | 2006 | 24.1 | 23.5 | 24.7 |
| Younger than 75 Years | 2007 | 22.6 | 22.1 | 23.2 |
| Younger than 75 Years | 2008 | 22.3 | 21.8 | 22.8 |
| Younger than 75 Years | 2009 | 22.4 | 21.9 | 22.9 |
| Younger than 75 Years | 2010 | 21.6 | 21 | 22.1 |
| Younger than 75 Years | 2011 | 21.6 | 21.1 | 22.1 |
| Younger than 75 Years | 2012 | 20.9 | 20.4 | 21.3 |
| Younger than 75 Years | 2013 | 21.6 | 21.1 | 22.1 |
| Younger than 75 Years | 2014 | 21.9 | 21.4 | 22.4 |
| Younger than 75 Years | 2015 | 22.4 | 21.9 | 22.9 |
| Younger than 75 Years | 2016 | 22.9 | 22.4 | 23.4 |
| Younger than 75 Years | 2017 | 23.3 | 22.9 | 23.8 |
| Younger than 75 Years | 2018 | 23.4 | 22.9 | 23.8 |
| Younger than 75 Years | 2019 | 23.6 | 23.1 | 24.1 |
| Older Than 75 Years | 1999 | 1588.5 | 1569.1 | 1607.8 |
| Older Than 75 Years | 2000 | 1577.7 | 1558.5 | 1596.8 |
| Older Than 75 Years | 2001 | 1554.1 | 1535.3 | 1572.9 |
| Older Than 75 Years | 2002 | 1550.5 | 1531.8 | 1569.2 |
| Older Than 75 Years | 2003 | 1563 | 1544.4 | 1581.6 |
| Older Than 75 Years | 2004 | 1535.5 | 1517.2 | 1553.8 |
| Older Than 75 Years | 2005 | 1553.9 | 1535.7 | 1572.2 |
| Older Than 75 Years | 2006 | 1500.7 | 1483 | 1518.4 |
| Older Than 75 Years | 2007 | 1508.3 | 1490.7 | 1525.8 |
| Older Than 75 Years | 2008 | 1522.3 | 1504.8 | 1539.7 |
| Older Than 75 Years | 2009 | 1501 | 1483.7 | 1518.2 |
| Older Than 75 Years | 2010 | 1525.3 | 1508 | 1542.5 |
| Older Than 75 Years | 2011 | 1524.3 | 1507.3 | 1541.3 |
| Older Than 75 Years | 2012 | 1513.2 | 1496.5 | 1530 |
| Older Than 75 Years | 2013 | 1538.6 | 1521.9 | 1555.4 |
| Older Than 75 Years | 2014 | 1527.5 | 1511 | 1544 |
| Older Than 75 Years | 2015 | 1519.7 | 1503.4 | 1536 |
| Older Than 75 Years | 2016 | 1479.3 | 1463.3 | 1495.2 |
| Older Than 75 Years | 2017 | 1475.2 | 1459.5 | 1491 |
| Older Than 75 Years | 2018 | 1433.9 | 1418.6 | 1449.2 |
| Older Than 75 Years | 2019 | 1425.8 | 1410.7 | 1440.9 |
